# Transcriptome and proteome characterization of surface ectoderm cells differentiated from human iPSCs

**DOI:** 10.1038/srep32007

**Published:** 2016-08-23

**Authors:** Ying Qu, Bo Zhou, Wei Yang, Bingchen Han, Yi Yu-Rice, Bowen Gao, Jeffery Johnson, Clive N. Svendsen, Michael R. Freeman, Armando E. Giuliano, Dhruv Sareen, Xiaojiang Cui

**Affiliations:** 1 Department of Surgery, Samuel Oschin Comprehensive Cancer Institute, Los Angeles, CA, 90048, USA; 2 Board of Governors-Regenerative Medicine Institute, Cedars-Sinai Medical Center, Los Angeles, CA, 90048, USA; 3 iPSC Core, The David and Janet Polak Foundation Stem Cell Core Laboratory, Los Angeles, CA, 90048, USA; 4 Department of Biomedical Sciences, Cedars-Sinai Medical Center, Los Angeles, CA, 90048, USA

## Abstract

Surface ectoderm (SE) cells give rise to structures including the epidermis and ectodermal associated appendages such as hair, eye, and the mammary gland. In this study, we validate a protocol that utilizes BMP4 and the γ-secretase inhibitor DAPT to induce SE differentiation from human induced pluripotent stem cells (hiPSCs). hiPSC-differentiated SE cells expressed markers suggesting their commitment to the SE lineage. Computational analyses using integrated quantitative transcriptomic and proteomic profiling reveal that TGFβ superfamily signaling pathways are preferentially activated in SE cells compared with hiPSCs. SE differentiation can be enhanced by selectively blocking TGFβ-RI signaling. We also show that SE cells and neural ectoderm cells possess distinct gene expression patterns and signaling networks as indicated by functional Ingenuity Pathway Analysis. Our findings advance current understanding of early human SE cell development and pave the way for modeling of SE-derived tissue development, studying disease pathogenesis, and development of regenerative medicine approaches.

The ectoderm, derived from the epiblast, differentiates into the central neural ectoderm and the surface ectoderm (SE). Lineage commitment of the surface ectoderm is regulated by bone morphogenetic protein (BMP) activity. SE, a single-layered epithelium originating from the lateral portions of the ectoderm, further differentiates to epidermis and other ectodermal appendages such as hair follicles, mammary glands, salivary glands and teeth. The molecular basis of ectodermal appendage development is not well understood. Studies have suggested that the specification of ectodermal appendages from SE cells largely depends on the microenvironment^1^. Known signaling pathways responsible for further differentiation from SE include BMP^2^, Wnt/β-catenin, ectodysplasin (Eda)/NF-κB, fibroblast growth factor (Fgf), Hedgehog, and transforming growth factor β (TGFβ) pathways^3^,^4^,^5^. Notably, the downstream SMAD1 activity of BMP signaling is stabilized by Wnt/GSK^6^.

Most tissues and organs derived from SE are exposed to the external environment and are vulnerable to environmental damages. Regenerative medicine in this area holds great promise in tissue repair and bioengineering, but still requires further understanding of early development at the molecular level. BMP signaling is known to play important roles in neural and epidermal fate determination as shown in previous studies^7^,^8^. BMP4 protein is capable of inducing epidermal and other ectodermal organ differentiation and inhibiting neural differentiation^9^. Of note, BMP4 acts in concert with γ-secretase, a multi-subunit membrane-associated protease complex, to program this developmental process. The cleavage of E-cadherin and Notch by γ-secretase induces non-neural ectoderm, later generates surface ectoderm, and inhibits neural ectoderm commitment^10^,^11^. Although the γ-secretase inhibitor (N-[(3,5-Difluorophenyl)acetyl]-L-alanyl-2-phenyl]glycine-1,1-dimethylethyl ester) (DAPT) was not required for the commitment of non-neural ectoderm, it does inhibit mesodermal differentiation in response to BMP4^12^. Thus, the combination of BMP4 and DAPT, was applied to induce the formation of human SE progenitors from human embryonic stem cells (hESCs)^12^.

Induced pluripotent stem cells (iPSCs) can be generated directly from terminally differentiated cells^13^. They can bypass the need for embryos and can be generated in a patient-specific manner, opening up an avenue for personalized regenerative medicine. Human iPSCs (hiPSCs) have been successfully induced to generate multiple cell types such as neurons, cardiomyocytes, and hepatocytes^14^,^15^,^16^. These unlimited supplies of autologous cells could be used to generate transplants without the risk of immune rejection. The iPSC technology can also be used for disease modeling and drug development^17^. Differentiation of iPSCs to SE cells is the first step of realizing personalized regenerative medicine to reduce hair loss and to treat diseases related to SE-derived tissues, such as limbal stem cell deficiency that can lead to visual impairment and blindness^18^ and epidermolysis bullosa that causes blisters in the skin and mucosal membranes ranging in severity from mild to lethal^19^,^20^,^21^. However, a reliable and efficient protocol for differentiating hiPSCs into SE has not yet been reported.

In this study, we tested whether the combination of BMP4 and DAPT, which is able to induce SE from hESCs^12^, induces SE differentiation of hiPSCs. We further used cDNA microarray and quantitative proteomic analyses to characterize the molecular basis of SE differentiation. Importantly, we reveal that TGFβ signaling plays a critical role in SE differentiation and TGFβ-RI inhibition-induced SE marker up-regulation.

## Results

### SE differentiation from hiPSCs

**Figure 1:**
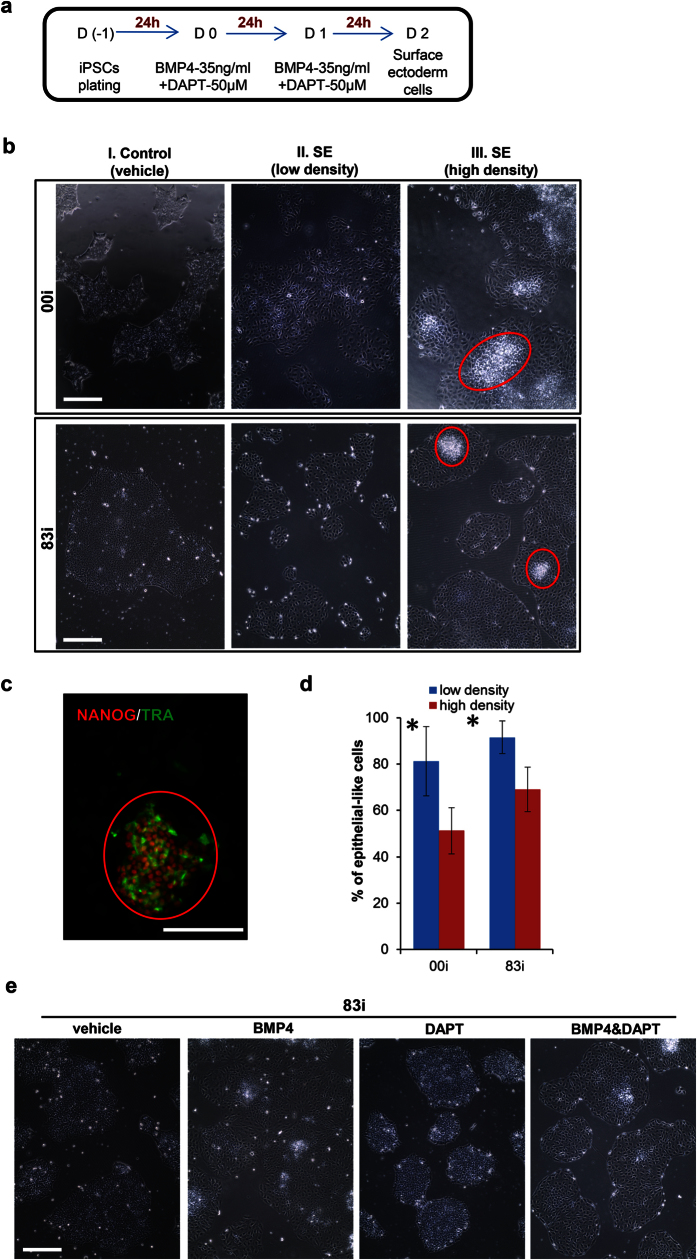
Induction of surface ectoderm (SE) differentiation from hiPSCs. (**a**) Protocol for *in vitro* differentiation of hiPSCs to SE. **(b**) Cell morphological changes after SE induction. The top and bottom panels show the morphological changes in undifferentiated and differentiated hiPSC lines 00i and83i, respectively. Red circles: undifferentiated hiPSCs. Original magnification x100. **(c**) Representative immunofluorescence image shows undifferentiating cells under high density condition expressing pluripotent markers NANOG and TRA-1-81. Original magnification x200. (**d**) Percentile of epithelial-like (differentiated) after 48 h SE induction in 00i and 83i hiPSCs. hiPSCs were induced to SE differentiation in low or high density. After 48 h induction, number of epithelial-like and iPSCs-like morphologies was counted under high magnification (x200) from five random fields. % of epithelial-like cells was calculated as following: % = (number of epithelial-like cells/total number of cells) x100 and plotted as (mean ± SD). *p < 0.05. **(e**) Representative images displayed cell morphologies treated by vehicle (DMSO), BMP4, DAPT or (BMP4 + DAPT) after 48 h. Original magnification x100. Bars: 100 μm.

SE cells, originating from intraembryonic ectoderm cells, are the stem cells for all the epidermal appendages such as skin, nail, hair, mammary gland, eye, and ear. Given that BMP4 induces SE differentiation from hESCs, and its effect can be enhanced by the γ-secretase inhibitor DAPT^22^, we tested whether this protocol could also induce SE differentiation from well-characterized 83i and 00i hiPSC lines, which were generated from normal human fibroblast cells at the Cedars-Sinai Medical Center iPSC Core^15^,^20^. hiPSCs at high (6 × 10^4^/cm^2^, 60% confluency) and low (3 × 10^4^/cm^2^, 30% confluency) density were plated and subsequently treated with BMP4 (35 ng/ml) and DAPT (50 μM) (Fig. 1a). We initially evaluated the optimal starting cell density for SE differentiation^23^,^24^. Fresh medium was changed every 24 h for a 48 h period. Compared with vehicle-treated hiPSCs (Fig. 1b,I), the morphology of hiPSCs after the combination treatment transitioned from small, tightly packed cells to enlarged and flattened epithelial morphology, but still within a distinct colony boundary, indicating cell differentiation in both low (Fig. 1b,II) and high (Fig. 1b,III) density conditions. There were undifferentiated hiPSC clusters in the center of the colonies from initial high density plating (Fig. 1b,III). These cell clusters retained pluripotency markers NANOG and TRA-1-81expression (Fig. 1c). Notably, in low density plates, hiPSCs were differentiated into nearly homogenous cobblestone epithelial morphology with few dense clusters (Fig. 1b,II). A lower density of hiPSC plating was associated with significant increased percentages of epithelial-like cells in both 83i and 00i hiPSC lines (Fig. 1d). This overt phenotypic change was likely dictated by BMP4^25^ and not by DAPT that has a role in inhibition of mesodermal inhibition not commitment of SE fate^12^ (Fig. 1e). In summary, BMP4 and DAPT can induce hiPSCs to display an epithelial morphology.

### Identification of SE-related markers and signaling pathways

A molecular understanding of human SE cells is currently limited as there have been no studies of gene expression. To identify potential human SE markers, we searched the gene profiling databases across different non-human species from previous studies. Analysis of the LifeMap Discovery database (http://discovery.lifemapsc.com/) showed that the selective SE markers for mouse SE cells were *Foxg1, Krt18, Krt8*, and *P63* (Supplementary Table 1, red color). Mouse SE cells did not express *Pax6* (Supplementary Table 1, blue color)^26^. BMP4 (Supplementary Fig. 1a)^27^, P63 (Supplementary Fig. 1b)^28^, and Mir450b (Supplementary Fig. 1c)^26^ were the upstream regulators for inducing SE differentiation. In response to BMP4, intraembryonic ectoderm cells exhibited increased levels of p-SMAD1/5/9 and its downstream genes *Dlx5, Gata2, Msx1, Tbx3*, and *Msx2*^29^,^30^,^31^,^32^ (Supplementary Fig. 1a). P63 initiated the expression of SE markers including *Krt18, Krt8, Krt14, AP-2γ*, and Perp^33^ (Supplementary Fig. 1b), while Mir450b inhibited the expression of *Pax6* and thus promoted epidermal specification from SE cells (Supplementary Fig. 1c)^26^.

**Figure 2:**
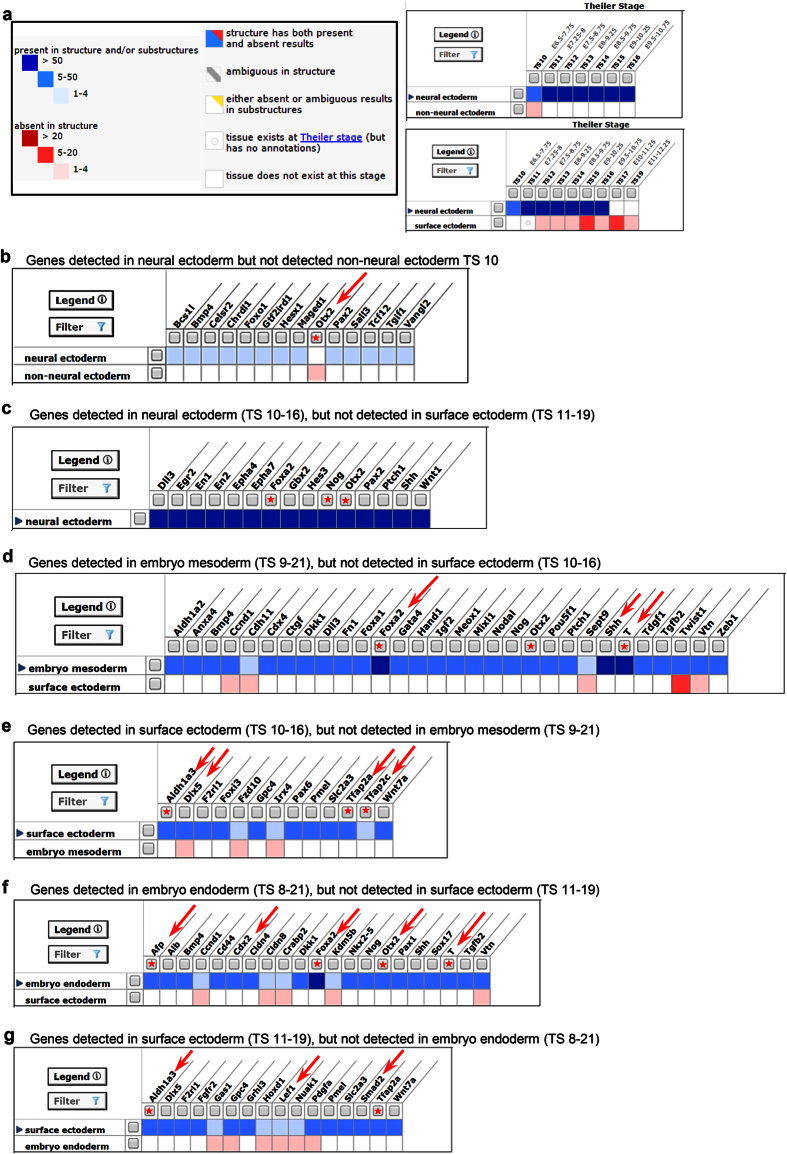
MGI database analysis of non-neural/surface ectoderm markers. (**a**) Summarize the co-existence of non-neural ectoderm, neural ectoderm and neural ectoderm in mouse embryo Theiler stages (TS). Graphs show the markers the greatest differential expression in neural vs. non-neural **(b**), neural vs. surface ectoderm **(c)**, embryo mesoderm vs. surface ectoderm **(d**), surface ectoderm vs. embryo mesoderm (**e**), embryo endoderm vs. surface ectoderm **(f**), and surface ectoderm vs. embryo endoderm **(g**). Red stars: selected markers used in the following experiments.

We next searched a Mouse Genome Informatics (MGI) Web Site (GXD, http://www.informatics.jax.org/expression.shtml) to explore genes differentially expressed between SE, neural ectoderm (NE), embryonic mesoderm, and embryonic endoderm^34^. As indicated in Fig. 2a, non-NE and NE co-exist at the theiler stage 10, while SE and NE co-exist from theiler stage 11 to 16. However, there are no data on theiler stage 11 for SE, so we compared data from theiler stage 12. At theiler stage 10, *Otx2* was expressed only in mouse embryonic NE (Supplementary Table 2, Fig. 2b)^35^. We found 15 most highly expressed genes (over 50 fold) in neural but not in surface ectoderm such as *Foxa2, Nog*, and *Otx2* (Fig. 2c). Likewise, we compared SE with embryonic mesoderm (Fig. 2d,e) and embryonic endoderm (Fig. 2f,g). Genes such as *Foxa2, Gata4, Brachyury* (*T*)*, Pou5f1* (*Oct3/4*), *Afp*, *Cdx2*, and *Otx2* were expressed in embryonic mesoderm and endoderm. On the contrary, genes such as *Tfap2a, Tfap2c, Aldh1a3*, *Dlx5*, and *Lef1* were only expressed in SE.

Upon querying LifeMap and MGI databases, a panel of markers and signaling pathways were identified in differentiating neural and non-neural/surface ectoderm, embryonic mesoderm, and endoderm cells, the majority of which were from mouse gene expression studies. Due to scant knowledge on the molecular biology of human SE cells, we selected KRT8, KRT18, KRT19, P63, AP-2γ, AP-2α, ALDH1A3, CDH1, and Desmoplakin as markers for our study on SE differentiation from hiPSCs.

### hiPSC-derived SE cells express SE markers derived from databases analysis

**Figure 3:**
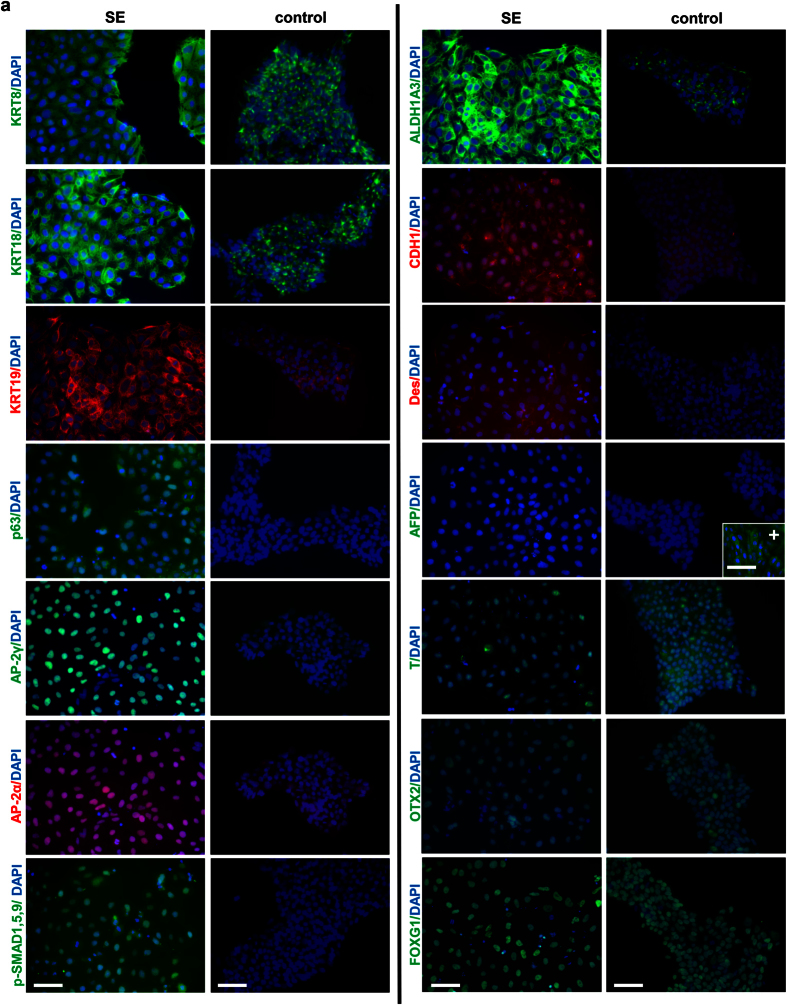
Immunofluorescence staining of selected markers in SE and hiPSCs. SE markers KRT8, KRT18, KRT19, p63, AP-2α, AP-2γ, ALDH1A3, p-SMAD1/5/9, CDH1, Desmoglein 3 and FOXG1 endodermal marker AFP, Mesodermal marker Brachyury (T), and neural ectoderm marker OTX2 were stained. DAPI was used to stain nuclei. The secondary antibodies Alexa −488 and Alexa −594 were used to visualize green and red signals, respectively. Insert in AFP staining image (+): Hela control cells stained by anti-AFP antibody and showed positive staining. Original magnification x200. Bars: 50 μm.

To confirm that the BMP4 + DAPT treatment gives rise to SE differentiation, we used immunofluorescence staining to examine the expression of SE markers selected from combined analysis of LifeMap and MGI databases. Transcription factors AP-2α^36^, P63^37^,^38^ and AP-2γ^22^,^39^ were selected to identify SE cells. Other cell surface markers for SE cells included KRT8, KRT18, KRT19, CDH1, and Desmoplakin^40^,^41^. hiPSC-derived SE cells highly expressed above-mentioned SE markers. Although hiPSCs expressed moderate levels of KRT8, 18, and 19, SE cells showed much higher levels of these proteins as assayed by western blotting (Supplementary Fig. 2a). As BMP4 is the key factor inducing SE differentiation, we also checked the levels of its downstream effector p-SMAD1/5/9 to verify the activation of BMP4 signaling. In contrast to undifferentiated hiPSCs as a control, p-SMAD1/5/9 level was significantly increased in putative human SE cells, while the levels of neural ectoderm marker OTX2 were not increased (Fig. 3). In agreement, expression of early pan-mesoderm marker Brachyury (T) and endoderm marker AFP was not elevated in SE cells compared to hiPSCs (Fig. 3). hESCs showed similar cell morphological changes as hiPSCs upon BMP4 and DAPT treatment (Supplementary Fig. 2b). These hESC-derived SE cells showed similar marker expression pattern as hiPSCs (Supplementary Fig. 2c). Of note, a known mouse SE marker Foxg1^42^ was found to be expressed in both hiPSCs and SE cells. We reasoned that although Foxg1 is an SE marker in mouse embryonic tissues, it might not be related to human SE development. We found that FOXG1 was also highly expressed in hESCs; and interestingly, its levels dropped significantly when SE differentiation was induced (Supplementary Fig. 2c). This is also supported by a recent report on the role of Foxg1 in neural differentiation^43^. In summary, no significant induction of well-known neural ectoderm, mesoderm, and endoderm markers expression was observed in SE cells. These data suggest that BMP4 and DAPT treatment induces the differentiation of hiPSCs selectively towards SE cells.

### Transcriptome and proteome characterization of hiPSC-derived SE cells

**Figure 4:**
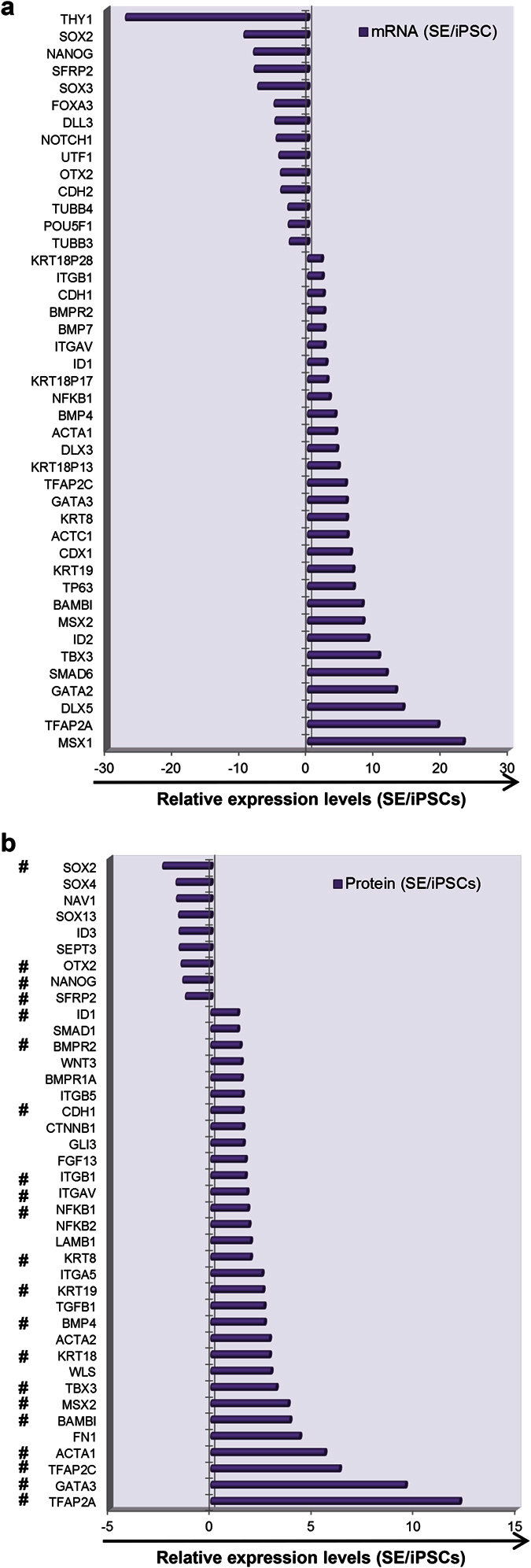
Characterization of SE cells using cDNA microarray and quantitative proteomics analysis. Top ranked up- and down-regulated genes analyzed by cDNA microarray **(a**) and quantitative proteomics analyses **(b**). cDNA microarray and quantitative proteomics analysis were performed using SE and hiPSCs. Up- or down-regulation of mRNA or protein levels were calculated via comparing SE with hiPSCs. Relative expression ratios of molecule expression levels in SE vs. hiPSCs are plotted. #: genes whose expression change are consistent in both mRNA and protein levels (SE/iPSCs).

To date, there are no transcriptome or proteome profiles of human SE cells, although there is limited gene expression data from mouse^34^ and chicken^44^. To explore the molecular basis of hiPSC differentiation into SE cells, we applied cDNA microarray analysis and quantitative proteomics to compare differentially expressed mRNA and proteins in hiPSCs and derived SE cells. As shown in Fig. 4a, mRNA levels of SE markers such as *KRT8*, *KRT19*, *KRT18*, *AP-2α*, *AP-2γ*, *MSX1*, *MSX2*, and *P63* were dramatically increased in SE cells compared with control hiPSCs, whereas levels of pluripotent stem cell markers such as *SOX2, NANOG*, *POU5F1* (*OCT3*/*4*), *UTF1*, and *THY1* were down-regulated. Genes in the BMP signaling network, such as *BMPR*, *ID1*, *ID2*, and *TBX3*, were also up-regulated in SE cells. In addition, expression of neural ectoderm (*DLL3, PAX6*, *OTX2*, *SOX3*), pan-mesoderm (*Brachyury* (*T*), *FOXC1*), and endoderm lineage (*FOXA1*, *CDX2*) markers was not induced. Relative expression levels of selected up- and down-regulated genes were confirmed by qRT-PCR in both hiPSC (Supplementary Fig. 3a, left) and hESC (Supplementary Fig. 3a, right) groups.

We next compared 72 h (3d-SE) or 48 h (2d-SE) SE induction to determine whether 48 h induction was sufficient by comparing mRNA profiles of 2d- and 3d-SE cells. These cells display a similar morphology (Supplementary Fig. 3b). We employed Ingenuity Pathway Analysis (IPA) online software to compare 3d-SE and 2d-SE cDNA microarray datasets. IPA is a well-known tool for predicting the signals that might cause the gene expression changes on input data. Three analyses including Canonical pathways (to compare well-established signaling pathways), Bio Functions (to compare activation or inhibition of critical biological processes or functions) and Upstream Regulators (to compare predicted molecules or signals upstream of the observed gene expression changes) were performed (see Methods section for detailed description). Comparison on Canonical Pathways (R = 0.7727), Bio Functions (R = 0.7152) and Upstream Regulators (R = 0.9382) exhibited significant positive correlations between 2d-SE and 3d-SE (Supplementary Fig. 3c-e). Together, from IPA bioinformatics analysis, it appears that 48 h of BMP4 + DAPT induction was sufficient in patterning hiPSCs towards SE fate.

Lastly, we conducted quantitative proteomic analysis to expand the abovementioned 2d-SE mRNA profile. Consistent with the mRNA expression results, the pluripotent markers SOX2 and NANOG were down-regulated and SE markers such as TFAP2C, TFAP2A, GATA3, MSX2, KRT 8, KRT18, KRT19 were up-regulated in SE cells (Fig. 4b, # labeled genes). Of note, actin family genes ACTA1, ACTA2 and ACTC1 were up-regulated in SE cells (mRNA and protein). These genes are involved in TGFβ1-mediated signaling that is important for skeleton muscle differentiation in human SE (or mesoderm) development^45^,^46^. To consolidate our study, we compared ectoderm marker expression in SE cells and NE cells. NE cells were induced from hiPSCs using the commercial StemXVivo kit (Supplementary Fig. 3f). NE cells expressed higher levels of neural ectoderm markers OTX2, TUJ1, and SOX11 but not SE markers AP-2α, AP-2γ and KRT18 relative to SE cells (Supplementary Fig. 3g). These data further demonstrate that SE cell differentiation from hiPSCs can be confirmed using gene expression profiling.

### Pathway analysis of hiPSC-derived SE cells

**Figure 5:**
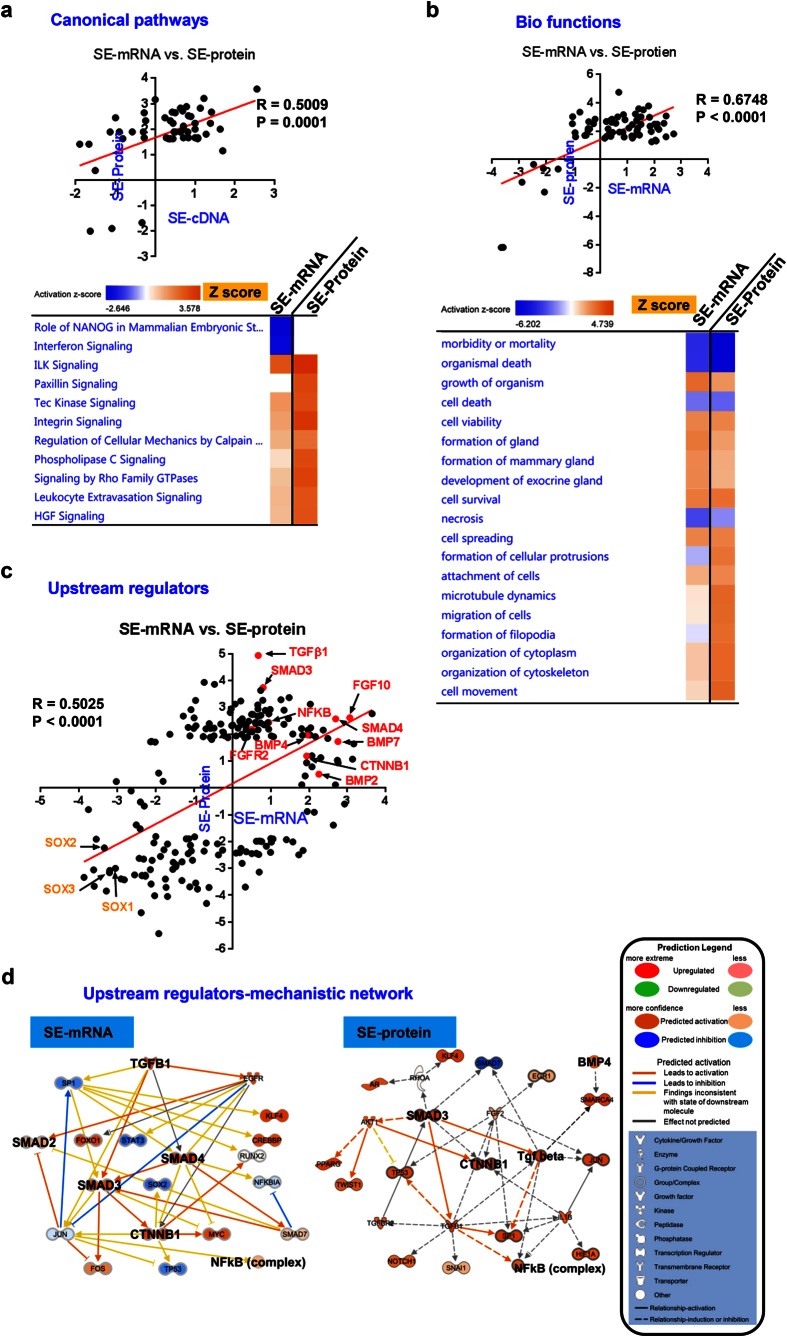
Functional analysis of SE cells using IPA software. IPA was performed to analyze the SE-mRNA and SE-protein datasets as shown in Fig. 4. (**a**) Canonical pathway analysis shows most significant up- and down-regulated canonical pathways in SE cells compared to hiPSCs. Pearson’s correlation coefficient (top) and activation z-score (bottom) are shown. **(b**) Bio function analysis using IPA shows most significant up- and down-regulated bio-functions in SE cells compared to hiPSCs. Pearson’s correlation coefficient (top) and activation z-score (bottom) are shown. (**c**) Upstream Regulator Analysis in IPA is a tool that predicts upstream regulators from gene expression data based on the literature and compiled in the Ingenuity Knowledge Base. A Fisher’s Exact Test p-value is calculated to assess the significance of enrichment of the gene expression data for the genes downstream of an upstream regulator. X-axis and Y-axis showed the upstream regulators in SE vs. hiPSCs using cDNA microarray (SE-mRNA) and quantitative proteomics (SE-protein) datasets, respectively. Pearson’s correlation coefficient was calculated. **(d)** Mechanistic network analysis shows the activation of TGFβ superfamily, Wnt/β-catenin, and NF-κB signaling pathways in both cDNA microarray (left) and quantitative proteomics (right) datasets.

To gain further insight into the biological pathways involved in SE differentiation, we performed IPA of our cDNA microarray (SE-mRNA) and proteomics (SE-protein) datasets. First, we performed three independent analyses, Canonical Pathways, Bio Functions and Upstream Regulators, to evaluate the consistency between two datasets. A statistically significant correlation (R = 0.5009, P = 0.0001) between SE-mRNA and SE-protein datasets was observed in canonical pathway analysis (Fig. 5a, top). The most down-regulated pathways were stem cell and interferon signaling, while the most up-regulated pathways were related to cell-matrix interaction and cytoskeleton activity (Fig. 5a, bottom), which were consistent with the cell morphological changes in hiPSCs differentiation to SE cells (see Fig. 1c). Similarly, analysis on Bio Functions also indicated a positive correlation (R = 0.6748, P < 0.0001) between mRNA and protein datasets (Fig. 5b, top). The most down-regulated Bio Functions were related to death signaling, while the most up-regulated Bio Functions of cytoskeleton re-organization were related to differentiation and survival (Fig. 5b, bottom). We next sought to determine whether these Canonical Pathways and Bio Functions were enriched in NE cells induced by the StemXVivo kit. By comparing 2d-SE and 3d-SE cDNA microarray datasets, some Canonical Pathways up-regulated in SE cells (Z score, shown in red) and some were down-regulated in NE cells (Z score, shown in blue) (Supplementary Fig. 4a). Furthermore, these two cell types displayed distinct Bio Functions profiles (Supplementary Fig. 4b).

We next conducted IPA Upstream Regulator analysis of our datasets to uncover critical upstream signals governing gene expression profiles in SE differentiation. Notably, there was a positive correlation (R = 0.5025, P < 0.0001) in identified signals from transcriptomics and proteomics datasets (Fig. 5c). Prominent up-regulated signals include TGFβ superfamily signaling components including BMP4, TGFβ1, and SMAD, while SOX family was among the most significantly inhibited regulators (Fig. 5c). It is noteworthy that these most activated regulators as shown in Fig. 5c in SE cells (*BMP7, TGFβ1, SMAD3, SMAD4, CTNNB1, NF-κB*) were inhibited in NE cells (Supplementary Fig. 4c). Lastly, we constructed a Mechanistic Network to reveal the intrinsic connections within highlighted regulators in Fig. 5c based on the existing knowledge from IPA software. Consistently, the Mechanistic Network revealed the crosstalk among TGFβ/SMAD/CTNNB1/NF-κB pathways in both mRNA and protein profiling of SE cells (Fig. 5d). In stark contrast, NE cells exhibited decreased TGFβ1/SMAD/CTNNB1/NF-κB activity (Supplementary Fig. 4d). In summary, our data suggest that specific signaling pathways converging on TGFβ superfamily proteins are associated with SE differentiation.

### Inhibition of TGFβ-RI enhances SE differentiation-associated marker expression

**Figure 6:**
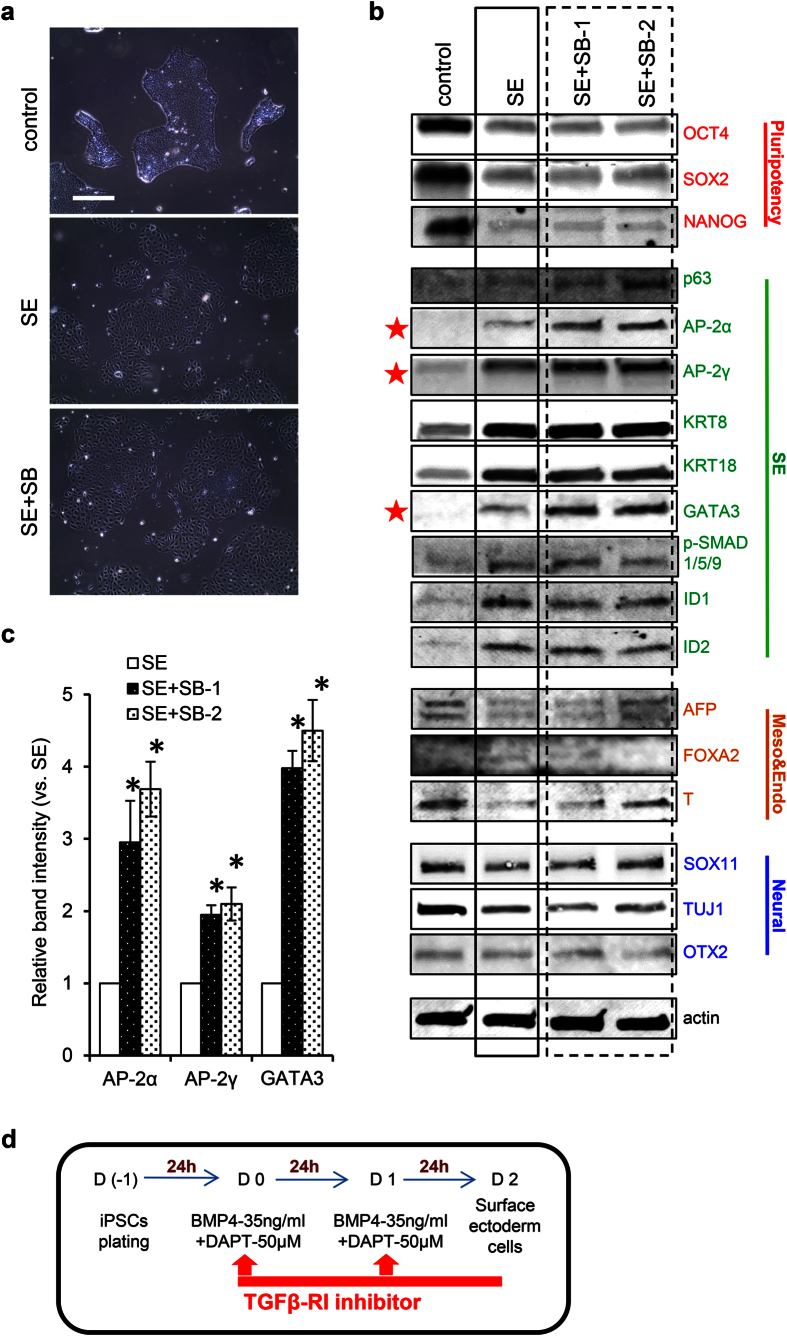
Selective inhibition of TGFβ-RI signaling enhances SE differentiation. (**a**) Cell morphologies in hiPSC (control), SE induction (SE, using BMP4 and DAPT), and SE induction with TGFβ-RI inhibitor SB431542 (two separate experiments named as SE + SB-1 and SE + SB-2) groups. Bar: 100 μm. Original magnification x100. (**b**) Immunoblotting analysis showed the expression of SE and other lineage markers in all groups. Actin was used as loading control. SE + SB-1 and SE + SB-2 suggested two separate experiments. Red stars: greatly altered compared to SE. Black box: results from SE group. Dashed black box: results from SE + SB groups. The gels have been run under the same experimental conditions. Full-length blots are shown in Supplementary Fig. 6. **(c**) Relative intensity is represented by selected marker expression in SE+SB groups compared to SE only group and plotted. Plotted data represent three independent experiments. *p < 0.05 (relative to SE groups). **(d**) Modified protocol for *in vitro* differentiation of hiPSCs to SE. We modified the existing protocol by adding TGFβ-RI signaling inhibitor to improve the SE differentiation.

Our findings showed that TGFβ1 signaling was significantly up-regulated in SE cells, which might in part be due to the negative feedback from BMP/SMAD signaling activation. Because activation of TGFβ1 signaling represses BMP signaling^47^, up-regulated TGFβ1 signaling in SE cells may reflect a negative feedback from BMP4 stimulation in iPSCs. We thus tested whether TGFβ-receptor I (RI) inhibition potentiates SE differentiation. To this end, SB431542, a specific inhibitor of TGFβ-RI signaling, was added to the culture for SE induction. Previous studies have shown that SB431542, as with BMP4, elicited a flattened morphology in iPSCs^48^. Addition of SB431542 did not alter cell morphology elicited by BMP4 (Fig. 6a). We then used western blotting to assess the effects of SB431542 on SE differentiation in two separate experiments (SE + SB-1 and SE + SB-2). Expression levels of markers suggesting the activation of BMP4 signaling (p-SMAD1/5/9, ID1, ID2) and pluripotency (OCT4, SOX2, NANOG) were not affected by SB (Fig. 6b). Notably, the expression levels of SE markers AP-2α (3-fold), AP-2γ (2-fold) and GATA3 (4-fold)^49^ were significantly increased in SB + SE (dashed black box) groups relative to SE (black box) alone (Fig. 6c).

To rule out potential off-target effects of the inhibitors, we further tested another two TGFβ-R inhibitors: LY2157299^50^ (a TGFβ-RI inhibitor) and LY2109761^51^ (a dual inhibitor targeting TGFβ-RI and TGFβ-RII) in inducing SE differentiation from hiPSCs. Similar to SB431542, adding LY2157299 did not change the SE morphologies (Supplementary Fig. 5a, left panel). In addition, it increased the levels of SE markers (Supplementary Fig. 5b). Interestingly, dual inhibitor LY2109761 still induced the epithelial-like morphological changes but did not induce SE marker expression. This might suggest that TGFβ-RII activity is required in SE differentiation. On the contrary, inhibiting BMP signaling by adding DMH1^52^ or LDN-193189^53^ blocked the SE differentiation as suggested by maintaining iPSC-like morphology (Supplementary Fig. 5a, left panel) and lacking in SE marker induction (Supplementary Fig. 5b, top panel). These observations were also shown in hESCs (Supplementary Fig. 5a right panel and 5b bottom panel). Our results suggest that selective inhibition of TGFβ-RI signaling can increase SE marker expression in SE cells differentiated from hiPSCs (Fig. 6d).

## Discussion

Both NE and SE are differentiated from a common bi-potent progenitor, the definitive ectoderm. The formation of SE occurs in response to BMP4 signaling^9^. No reported studies have examined the induction of SE cells from hiPSCs. Here we developed a protocol for this procedure and characterized induced SE cells using gene expression and protein profiling.

It was necessary to test whether the combination of BMP4 and DAPT treatment can induce hiPSCs differentiation to SE cells. BMP4 reportedly induces a set of transcriptional factors such as *AP-2α, AP-2γ*, and *GATA3* in early non-neural development^54^,^55^. As the knowledge on human SE markers is limited, we exploited the mouse LifeMap and MGI databases in order to identify SE markers. p63 is one of the earliest transcription factors expressed during epidermal specification^37^,^38^ and is associated with ectodermal appendage specification^4^,^56^. p63 directly up-regulates Ap-2γ *in vivo*^22^,^39^ in the commitment of non-neural ectoderm differentiation^57^ by inhibiting neural differentiation^22^. AP-2γ, AP-2α^36^ and GATA3^2^ transcriptional factors are SE cell markers. Other cell surface markers for SE cells consist of KRT8, KRT18, KRT19, CDH1, and Desmoplakin^40^,^41^. Through immunofluorescence staining, mRNA profiling, and protein profiling, we found that differentiated SE cells expressed these markers with minimal induction of neural ectoderm. Notably, down-regulation of the neural ectoderm marker OTX2 was also observed^35^ only in SE cells but not in hiPSC-differentiated NE cells. However, we observed the elevation of a group of genes such as ACTA1, ACTA2 and ACTC1 in both mRNA and protein levels, suggesting mesodermal differentiation. Together, our protocol directs SE differentiation and SE marker expression to a large extent, however mesodermal differentiation might also be induced, to a minor extent, in this 48 h induction window.

There is little data on gene and protein expression profiles of human SE cells and SE differentiation. To our knowledge, we believe this study represents the first attempt to use comprehensive transcriptome and proteome analysis to better understand the biology of derived human SE cells. Signaling pathways involving TGFβ superfamily, Wnt/β-catenin, and NF-κB signaling were the key pathways in SE differentiation as analyzed by IPA software. In contrast to SE cells, NE cells showed down-regulation of these signaling pathways, which were known to play critical roles in non-neural ectoderm commitment through integration with BMP signaling^27^,^58^,^59^,^60^. We also found that both Bio Functions and Canonical Pathways analysis showed cytoskeleton organization and cell mobility alterations. Our findings were consistent with previous findings that SMAD-independent BMP4 signals regulated cytoskeleton mediated cell mobility^25^ and adhesion pathways in rat stem cells^61^. We further used Upstream Regulator analysis provided by IPA to reveal the mechanisms of regulatory networks for SE differentiation. Again, in our mRNA and protein profiles, key factors directing the activation of these key pathways were found. Although there is no available human SE database to compare with, we have profiled and characterized induced SE cells based on currently available information. The fact that our findings were consistent with previous studies^1^,^62^ strongly suggested SE differentiation using this novel protocol.

The top activated upstream regulators predicted by IPA, TGFβ1, primarily activates SMAD2/3, induces mesodermal differentiation, and activates Actin family expression^45^,^63^. Consistent with previous findings, elevation of Actin family gene expression in induced SE cells was observed in both mRNA and protein levels. The signaling transduction of TGFβ superfamily can be divided into two panels, BMP-panel and TGFβ-panel^64^. Given that the ligands from both TGFβ and BMP panels were shown to be activated in SE cells, SE differentiation was thus hypothesized to be regulated by both panels. As previous study showed that TGFβ1 antagonized the BMP signaling pathway^65^ which was the dominant inducer of SE differentiation, we asked whether blocking signal transduction by TGFβ-panel might increase SE differentiation induced by BMP4. We hypothesized that blocking TGFβ-RI signaling may enhance SE differentiation using SB431542, which was reported to induce human ESCs differentiation^48^. Indeed, blockage of TGFβ-RI signaling increased SE marker expression. SB431542 may further endorse BMP signaling by inhibiting TGFβ-RI signaling.

In conclusion, our study confirms that combining BMP4 and DAPT treatment can induce human SE differentiation from hiPSCs and lays the groundwork by using hiPSCs to generate and model organs or tissues. Our study also provides insight into the biology of human SE differentiation. Further research is warranted to elucidate the role of identified key pathways in specifying the differentiation of SE from hiPSC. However, we present promising advances towards generating human iPSC-derived SE that can be used for the regenerative medicine-based treatment of various SE-related diseases.

## Methods

### Human iPSC culture

00iCTR-n2 and 83iCTR-n1 hiPSC lines were generated from healthy human fibroblast cell lines at Cedars-Sinai Medical Center^15^,^20^. The characterization and karyotyping were performed as previously described^15^. The iPSC lines were seeded on feeder-free system using BD Matrigel Matrix and maintained in chemically-defined mTeSR1 medium (Stem Cell Technologies Inc., Vancouver, Canada).

### Human iPSC differentiation

The induction of surface ectoderm cells was performed by adding BMP4 (35 ng/ml) and DAPT (50 μM) 24 h after plating^22^. Medium was changed every 24 h for a 48 h or 72 h period. SB431542 was purchased from Sigma. SB431542 (5 μM) was added with the culture for SE induction. Neural ectoderm differentiation was performed using StemXVivo Ectoderm kit (R&D systems, Minneapolis, MN) according to the manufacture’s instruction. Briefly, hiPSCs were plated 24 h prior to treatment by differentiation medium (base medium with supplements provided in the kit) for 96 h. Medium was changed every 24 h. The induction of ectoderm cells was verified by goat anti-human OTX2 (provided in the kit) using western blotting.

### Immunofluorescence staining

Cells were fixed with 4% paraformaldehyde, permeabilized with 0.5% Triton X-100 for 10 min, and blocked with 3% bovine serum albumin for 30 min at room temperature. After blocking, cells were incubated overnight at 4 °C with primary antibodies. Primary antibodies are NANOG, TRA-1-81 (1:250, Stem cells); KRT8, KRT18, KRT19, AP-2γ, FOXG1 (1:200, Abcam); ALDH1A3, AP-2α, AFP (1: 200, Santa Cruz); Brachyury (T) (1:50, Novus); P63 (1:100, Genetex); p-SMAD1/5/9 (1:200, Cell Signaling); CDH1, Desmoplakin (1:500, BD Biosciences). Alexa 594-conjugated secondary antibody (1:400, red, Molecular Probes, Eugene, OR) or an Alexa 488-conjugated secondary antibody (1:400, green, Molecular Probes) was used to visualize the staining. Following three washes with PBS, slides were mounted with the VECTASHIELD mounting medium (Vector Laboratories, Burlingame, CA). Prior to mounting, slides were incubated with 2 μM 4′,6-diamidino-2-phenylindole (DAPI) fluorescence (Molecular Probes) for 10 min at 37 °C to stain the nuclei. The fluorescence images were taken using the EVOS FL Auto Cell Imaging System fluorescence microscope (ThermoFisher Scientific, NY, USA).

### cDNA microarray

Total RNA from SE cells and hiPSCs was extracted using the RNeasy Mini kit (Qiagen, Valencia, CA, USA) according to the manufacturer’s instructions. RNA quantity and purity were assessed by measurement of OD260/280 using a NanoDrop ND-1000 spectrophotometer. Gene expression profiling was conducted using Illumina Human HT-12 v4 BeadChip. Direct Hybridization Assay was used and chips were scanned on the HiScan system. The biological replications of cDNA microarray analysis were three. Statistic significant of differentiated mRNA expressions between SE and hiPSC cells was examined by T-test. A p-value < 0.05 was considered significant.

### Quantitative proteomics analysis

Tandem mass tagging (TMT)^66^ was coupled with two-dimensional liquid chromatography tandem mass spectrometry (2D LC-MS/MS) to quantify protein expression changes at the proteome scale. Total protein was extracted from three replicates of SE cells and hiPSC controls and tryptically digested using the filter-aided sample preparation method^67^, in which ammonium bicarbonate was replaced with triethyl ammonium bicarbonate to avoid interfering TMT labeling. Tryptic peptides were labeled with amine-reactive six-plex tandem mass tags (TMT6plex) in parallel, merged into one sample, desalted using C18 spin columns, and concentrated in a SpeedVac (all from Thermo Scientific). The TMT-labeled peptides were fractionated into 9 fractions by high-pH reversed-phase liquid chromatography (RPLC) using a 10-cm Hypersil GOLD column on an Ultimate 3000 XRS system (all from Thermo Scientific). Fractionated peptides were separated by low-pH RPLC using a 50 cm EASY-Spray analytical column on an EASY-nLC 1000 system and analyzed by an LTQ Orbitrap Elite mass spectrometer (all from Thermo Scientific)^68^. Briefly, mass spectra were acquired in a data-dependent manner, selecting up to 15 most abundant precursor ions for higher-energy collision dissociation (HCD)^69^. To minimize precursor ion co-isolation and to increase reporter ion intensity, the isolation width and normalized collision energy were set as 1.5 and 40, respectively. The acquired raw data were searched against the human Uniprot database (released on 02/10/2015, containing 89,775 sequences) with Proteome Discoverer (v2.0) (Thermo Scientific), using the SEQUEST algorithm^70^. Searching parameters were as follows: trypsin, up to two missed cleavage; precursor ion tolerance of 10 ppm; fragment ion tolerance of 0.02 Da; carbamidomethylation of cysteines and TMT6plex modification of lysines and peptide N-term as fixed modifications; acetylation of protein N-term, oxidation of methionines and deamidation of asparagines and glutamines as variable modifications. A stringent 1% false discovery rate was set to filter peptide identification. For protein quantitation, peptides with >30% precursor ion interference were excluded. Protein ratios were normalized against the median ratios, with the assumption that most proteins were not significantly changed before and after iPSC differentiation. To identify differentially expressed proteins, only proteins quantified by at least two peptides were analyzed. The biological replications of quantitative proteomic analysis were three. Statistic significant of differentiated protein expression between SE and hiPSC cells was examined by T-test. A p-value < 0.05 was considered significant.

### Western blotting

Proteins were extracted from human breast cancer cells using RIPA lysis buffer (Sigma- Aldrich) and protein concentration was determined by the BCA Protein Assay Kit (Thermo). Proteins (20 μg) were separated on 4–20% gradient gels and transferred onto PVDF membranes using Trans-Blot Turbo transfer pack (Bio-Rad) and Trans-Blot Turbo transfer system (Bio-Rad). Membranes were blocked in Odyssey blocking buffer (LI-COR) and incubated with primary antibodies overnight at 4 °C. The primary antibodies were AFP, AP-2α, GATA3, ID1, ID2, and Actin (Santa Cruz); FOXA2, AP-2γ, SOX11, KRT8, and KRT18 (purchased from Abcam); Brachyury (T) (Novus); P63 (Genetex); OCT4, SOX2, and NANOG (purchased from Stem cells); p-SMAD1/5/9 (purchased from Cell Signaling); OTX2 (R&D Bioscience); TUJ1 (Promega Inc). Primary antibodies were used at 1:1000 dilution overnight at 4 °C. The membranes were then incubated with IRDye 680CW or IRDye 800CW secondary antibodies (1:50000, LI-COR) for 1 h at room temperature. The membranes were scanned using the Odyssey infrared imaging system (LI-COR).

### Pathway and network analysis by IPA software

Analytics tools “Canonical Pathways”, “Bio Functions”, “Upstream Regulator Analysis” and “Mechanistic Networks” were available in Ingenuity Pathway Analysis software (IPA, QIAGEN Redwood City, CA, USA). Canonical Pathways analysis was to determine the most significant affected pathways. Bio Functions analysis was to predict the downstream biological processes which were increased or decreased based on input data. The Upstream Regulator analysis was based on prior knowledge of expected effects between transcriptional regulators and their target genes stored in the Ingenuity Knowledge Base. The analysis examined how many known targets of each transcription regulator are present in the user’s dataset, and also compares their direction of change (i.e. expression in the experimental sample(s) relative to control) to what was expected from the literature in order to predict likely relevant transcriptional regulators. If the observed direction of change was mostly consistent with a particular activation state of the transcriptional regulator (“activated” or “inhibited”), then a prediction was made about that activation state. Comparison analysis between input datasets was to visualize trends and similarities. The activation z‐score was used to infer likely activation states of upstream regulators based on comparison with a model that assigns random regulation directions. All analyses were carried out with differentially expressed genes (SE/iPSCs or NE/iPSCs). Differentially expressed genes/proteins were imported with the following cut-offs applied: fold change ≥1.5 and ≤ −1.5 as well as t-test p-value < 0.05.

### Statistical analysis

Values represent mean ± standard deviation (SD) of samples measured in triplicate. Quantitative data were analyzed using the Student’s t test and two-tailed distribution. Correlations between groups were analyzed by calculating the Pearson’s correlation coefficient (r) using the IBM SPSS statistics 20.0 program. Log-rank tests were performed to determine statistical significance. A p-value < 0.05 was considered significant.

## Additional Information

**How to cite this article**: Qu, Y. *et al*. Transcriptome and proteome characterization of surface ectoderm cells differentiated from human iPSCs. *Sci. Rep*. **6**, 32007; doi: 10.1038/srep32007 (2016).

## Supplementary Material

Supplementary Information
