# Supplementary material for: Transcriptome and proteome characterization of surface ectoderm cells differentiated from human iPSCs
Source: Sci Rep. 2016 Aug 23;6:32007. doi: 10.1038/srep32007 (PMC4994084; doi:10.1038/srep32007)
Supplement: Supplementary Information [file srep32007-s1.pdf]

Title: Transcriptome and proteome characterization of surface ectoderm cells differentiated from human iPSCs

Authors: Ying Qu[1], Bo Zhou[1], Wei Yang[1], Bingchen Han[1], Yi Yu-Rice[1], Bowen Gao[1], Jeffery Johnson[1], Clive N. Svendsen[2], Michael R. Freeman[1], Armando E. Giuliano[1], Dhruv Sareen[2,3,4], Xiaojiang Cui[1]

Supplementary Information

Supplementary Material and Methods

Human embryonic stem cell (hESC) culture

hESCs were seeded on feeder-free system using BD Matrigel Matrix and maintained in chemically-defined mTeSR1 medium (Stem Cell Technologies Inc., Vancouver, Canada).

qRT-PCR

Total RNA was extracted using the RNeasy Mini Kit (Qiagen) according to the manufacturer's instructions. Reverse transcription was done using the QuantiTect Reverse Transcription Kit. The qRT-PCR assay was done using an iCycler iQ Real-Time Thermocycler (Bio-Rad Laboratories) as described and GAPDH was used as an internal control. The primers used in this study were listed in Supplementary Table 3. Fold change was calculated according delta delta Ct value. Bar graphs represent mean ± SD (standard deviation) of three independent experiments.

Chemicals

BMP type I receptor inhibitor LDN-193189, non-selective BMP receptor inhibitor DMH1, TGFβ receptor type I/II inhibitor LY2109761, and TGFβ receptor type I inhibitor LY2157299 were

purchased from Cayman Chemical (Ann Arbor, MI, USA). The working concentration for each inhibitor was 500 nM, 1 μM, 2 μM, and 1 μM, respectively [1-3].

References

1 Horbelt, D. *et al.* Small molecules dorsomorphin and LDN-193189 inhibit myostatin/GDF8 signaling and promote functional myoblast differentiation. *J Biol Chem* **290**, 3390-3404, doi:10.1074/jbc.M114.604397 (2015).

2 Neely, M. D. *et al.* DMH1, a highly selective small molecule BMP inhibitor promotes neurogenesis of hiPSCs: comparison of PAX6 and SOX1 expression during neural induction. *ACS Chem Neurosci* **3**, 482-491, doi:10.1021/cn300029t (2012).

3 Yamasaki, A. *et al.* Identification of the role of bone morphogenetic protein (BMP) and transforming growth factor-beta (TGF-beta) signaling in the trajectory of serotonergic differentiation in a rapid assay in mouse embryonic stem cells in vitro. *J Neurochem* **132**, 418-428, doi:10.1111/jnc.12999 (2015).

Supplementary Figure Legends

Supplementary Figure 1. LifeMap online database analysis of upstream signals and downstream effectors. P63 **(a)** and BMP4 **(b)** are the upstream regulators directing SE differentiation, while Mir450b **(c)** is the upstream regulator inhibiting neural ectoderm differentiation from intraembryonic ectoderm cells.

Supplementary Figure 2. SE differentiation from human embryonic stem cell (hESC) line H9. (a) Western blotting analysis of KRT8, 18, and 19 expression in control hiPSCs and

differentiated SE cells. Actin was used as loading control. **(b)** Cell morphologies of control hESC line H9 and differentiated SE cells. Original magnification × 100. Bars: 100µm. **(c)** Immnoflourescence staining of SE markers KRT8, KRT18, KRT19, p63, AP-2α, AP-2γ, ALDH1A3, p-SMAD1/5/9, CDH1, Desmoglein 3 and FOXG1 endodermal marker AFP, Mesodermal marker Brachyury (T), and neural ectoderm marker OTX2 in control H9 and H9-derived SE cells. DAPI was used to stain nuclei. The secondary antibodies Alexa -488 and Alexa -594 were used to visualize green and red signals, respectively. Original magnification × 200. Bars: 50µm.

Supplementary Figure 3. Comparison of SE and NE differentiation at different time points. (a) qRT-PCR results of selected up- and down-regulated genes from cDNA microarray analysis. Relative gene expression levels (SE vs. hiPSCs or SE vs. hESCs, mean ± SD) are plotted. **(b)** Morphologies of SE induced from iPSCs at 48h and 72h time points are shown. Original magnification × 200. Bars: 100 µm. cDNA microarray analysis was conducted using iPSCs treated with vehicle (control), 48h SE induction (2d-SE), and 72h SE induction (3d-SE). Genes differentially expressed in 2d-SE vs. control and 3d-SE vs. control were identified. IPA was then employed to analyze the correlation of canonical pathways **(c)**, bio-functions **(d)**, and upstream regulators **(e)** between 2d-SE and 3d-SE. **(f)** Morphologies of iPSCs and NE cells are shown. Original magnification × 100. Bars: 100 µm. **(g)** Western blotting of neural and SE marker expression representing SE and neural differentiation. GAPDH was used as loading control. SE: surface ectoderm cells. C: vehicle control (iPSCs). N: NE differentiation.

Supplementary Figure 4. Comparison of SE cells and NE cells using IPA. (a) Canonical pathway analysis using IPA shows most significant up- and down-regulated pathways in SE compared to NE cells. Activation z-score (top) and –log (p-value) (bottom) are shown. **(b)** Bio-

function analysis using IPA shows most significant up- and down-regulated bio-functions in SE compared to NE cells. Activation z-score (top) and –log (p-value) (bottom) are shown. **(c)** Upstream Regulator Analysis in IPA is a tool that predicts upstream regulators from gene expression data based on the literature and compiled in the Ingenuity Knowledge Base. A Fisher's Exact Test p-value is calculated to assess the significance of enrichment of the gene expression data for the genes downstream of an upstream regulator. Dot plot showed the upstream regulators in SE (X-axis) vs. NE (Y-axis), respectively. Pearson's correlation coefficient was calculated. **(d)** Mechanistic network analysis showed the down-regulation of TGFβ superfamily, Wnt/β-catenin, and NF-κB signaling pathways in NE cells.

Supplementary Figure 5 Effects of inhibiting TGFβ superfamily activity in SE differentiation. (a) Cell morphologies in control, SE, and SE with inhibitors. hiPSCs and hESCs were treated with (BMP4+DAPT) or (BMP4+DAPT) and indicated inhibitors for 48h. Representative pictures are shown. Original magnification × 100. Bars: 100 μm. Left panel: hiPSCs. Right panel: hESCs. **(b)** Western blotting analysis of marker expression in control, SE and SE with inhibitors. Top panel: hiPSCs. Bottom panel: hESCs. Actin was used as loading control.

Supplementary Figure 6. Western blotting images for Fig. 6. The gels have been run under the same experimental conditions.

Supplementary Tables

Supplementary Table 1 SE-associated genes summarized in LifeMap Discovery Database

Species	Gene Symbol	Full Name	Expression	Localization	Method(s)	Validation
m	**Foxg1 (Bf1)**	**forkhead box G1**	**++**	intra		
m	**Krt18 (K18)**	**keratin 18**	**++**	intra	IS	P
m	**Krt8**	**keratin 8**	**++**	intra	IS	P
m	**Trp63 (P63)**	**transformation related protein 63**	**++**	intra	KO	RP
m	**Pax6**	**paired box 6**	**-**	intra		P
m	Aldh1a3 (RALDH3)	aldehyde dehydrogenase family 1, subfamily A3	+	intra	ISH, IS	RP
m	Arhgap31	Rho GTPase activating protein 31	+	intra	ISH, IS	RP
h	BMP2	bone morphogenetic protein 2	+	secreted	ISH	R
h	Bmp7 (OP1)	bone morphogenetic protein 7	+	secreted	ISH, IS	RP
m	BMP7	bone morphogenetic protein 7	+	secreted	ISH	R
ch	DLX5	distal-less homeobox 5	+	intra	IS	P
m	Epcam	epithelial cell adhesion molecule	+	surface		
m	Fgf10	fibroblast growth factor 10	+	secreted	ISH, IS	RP
m	Fgf8	fibroblast growth factor 8	+	secreted	ISH, IS	RP
m	Fgf9	fibroblast growth factor 9	+	secreted	ISH, IS	RP
m	Fgfr2 (Fgfr-2)	fibroblast growth factor receptor 2	+	surface	ISH, IS	RP
m	Fn1 (Fn-1)	fibronectin 1	+	secreted	ISH, KO	RP
m	Gata1	GATA binding protein 1	+	intra		
ch	GATA2	GATA binding protein 2	+	intra	IS	P
h	GRHL2	grainyhead-like 2 (Drosophila)	+	intra		
m	Grhl3	grainyhead-like 3 (Drosophila)	+	intra	ISH, IS	RP
h	GRHL3	grainyhead-like 3 (Drosophila)	+	intra		
m	Krt14 (K14)	keratin 14	+	intra	ISH, IS, Transgenic Mice	RP
m	Ltb	lymphotoxin B	+	surface	Transgenic Mice	RP
m	Mir450b	microRNA 450b	+		ISH	R
m	Msx1 (Hox7)	msh homeobox 1	+	intra	ISH, IS	RP
ch	MSX1	msh homeobox 1	+	intra	IS	P
ch	MSX2 (HOX-8)	msh homeobox 2	+	intra	IS	P
m	Nedd4	neural precursor cell expressed, developmentally down-	+	intra	ISH, IS	RP

		regulated 4				
m	Notch2	notch 2	+	surface	ISH, IS	RP
m	Otx2	orthodenticle homolog 2	+	intra	ISH, IS	RP
m	Perp	PERP, TP53 apoptosis effector	+	surface	KO	RP
m	Pmel	premelanosome protein	+	surface	ISH, IS	RP
m	Pth1r (Pthr1)	parathyroid hormone 1 receptor	+	surface	ISH, IS	RP
m	Smad1	SMAD family member 1	+	intra		P
m	Smad5	SMAD family member 5	+	intra		
m	Smad9 (Smad8)	SMAD family member 9	+	intra		
m	Sox2	SRY (sex determining region Y)-box 2	+	intra	ISH, IS	RP
m	Tfap2c (AP2gamma)	transcription factor AP-2, gamma	+	intra		
m	Zmiz1	zinc finger, MIZ-type containing 1	+	intra	ISH, IS	RP

Notes: ++, database–selected marker; -, undetected; +, expressed.
Intra: intra cellular; surface: cell surface
IS: immunostaining; ISH: in situ hybridization
P: protein; R: RNA

Supplementary Table 2 Differentially expressed genes in neural and non-neural ectoderm-GXD database analysis

MGI Gene ID	Gene Symbol	Gene Name	Assay Type	Age	Theiler Stage	Structure	Detected	PubMed ID
MGI:1914071	Bcs1l	BCS1-like (yeast)	Immunohistochemistry	E7.0	10	neural ectoderm	Yes	17049929
MGI:88180	Bmp4	bone morphogenetic protein 4	RNA in situ	E7.25	10	neural ectoderm	Yes	19393343
MGI:1858235	Celsr2	cadherin, EGF LAG seven-pass G-type receptor 2	RNA in situ	E6.75	10	neural ectoderm	Yes	11677057
MGI:1933172	Chrdl1	chordin-like 1	RNA in situ	E7.0	10	neural ectoderm	Yes	11118896
MGI:1890077	Foxo1	forkhead box O1	In situ reporter (knock in)	E7.0	10	neural ectoderm	Yes	21397048
MGI:1861942	Gtf2ird1	general transcription factor II I repeat domain-containing 1	Immunohistochemistry	E7.0	10	neural ectoderm	Yes	12971990
MGI:96071	Hesx1	homeobox gene expressed in ES cells	RNA in situ	E7.0	10	neural ectoderm	Yes	10882526
MGI:96071	Hesx1	homeobox gene expressed in ES cells	RNA in situ	E7.5	10	neural ectoderm	Yes	8565852
MGI:1930187	Maged1	melanoma antigen, family D, 1	Western blot	E7.0	10	neural ectoderm	Yes	12204258
MGI:97486	Pax2	paired box 2	RNA in situ	E7.5-8.0	10	neural ectoderm	Yes	7577673
MGI:109295	Sall3	sal-like 3 (Drosophila)	RNA in situ	E7.0	10	neural ectoderm	Yes	8798152
MGI:101877	Tcf12	transcription factor 12	RNA in situ	E7.0	10	neural ectoderm	Yes	15018808
MGI:1194497	Tgif1	TGFB-induced factor homeobox 1	RNA in situ	E7.0	10	neural ectoderm	Yes	16284942
MGI:2135272	Vangl2	vang-like 2 (van gogh, Drosophila)	RNA in situ	E7.0-7.5	10	neural ectoderm	Yes	11431695
MGI:97451	Otx2	orthodenticle homolog 2	In situ reporter (knock in)	E7.0	10	non-neural ectoderm	No	7588062

Supplementary Table 3 Primers used in this study

	Forward	Reverse
TFAP2C	CAGATGGACGAGGTGCAGAATG	GGGGAGGTTCAGAGGGTTCT
TFAP2A	GGAGGTCCCGCATGTAGAAG	CGACCCGGAACTGAACAGAA
KRT8	GCTGGTGGAGGACTTCAAGA	AGCTCCCGGATCTCCTCTTC
KRT18	GATCATCGAGGACCTGAGGG	ATCAATGACCTTGCGGAGCC
KRT19	CTACACGACCATCCAGGACC	TCCGTCTCAAACTTGGTTCG
OTX2	CCCGGTACCCAGACATCTTC	GCGGCACTTAGCTCTTCGAT
TUBB3	GAGACAGGTACAGGTCCACG	AGGCACGTACTTGTGAGAAGA
FOXA2	CGTTCCGGGTCTGAACTGT	CATGTTGCTCACGGAGGAGT
ACTA1	CACGATGTACCCTGGGATCG	GCCGATCCACACCGAGTATT
ID1	AATCATGAAAGTCGCCAGTG	ATGTCGTAGAGCAGCACGTTT
ID2	CCGTGAGGTCCGTTAGGAAA	GAGCTTGGAGTAGCAGTCGT
P63	TGGTGCGACAAACAAGATTG	ATAGGGACTGGTGGACGAGG
MSX2	TGGATGCAGGAACCCGG	AGGGCTCATATGTCTTGGCG
NFKB1	GCTTAGGAGGGAGAGCCCA	TATGGGCCATCTGTTGGCAG

Fig.S1

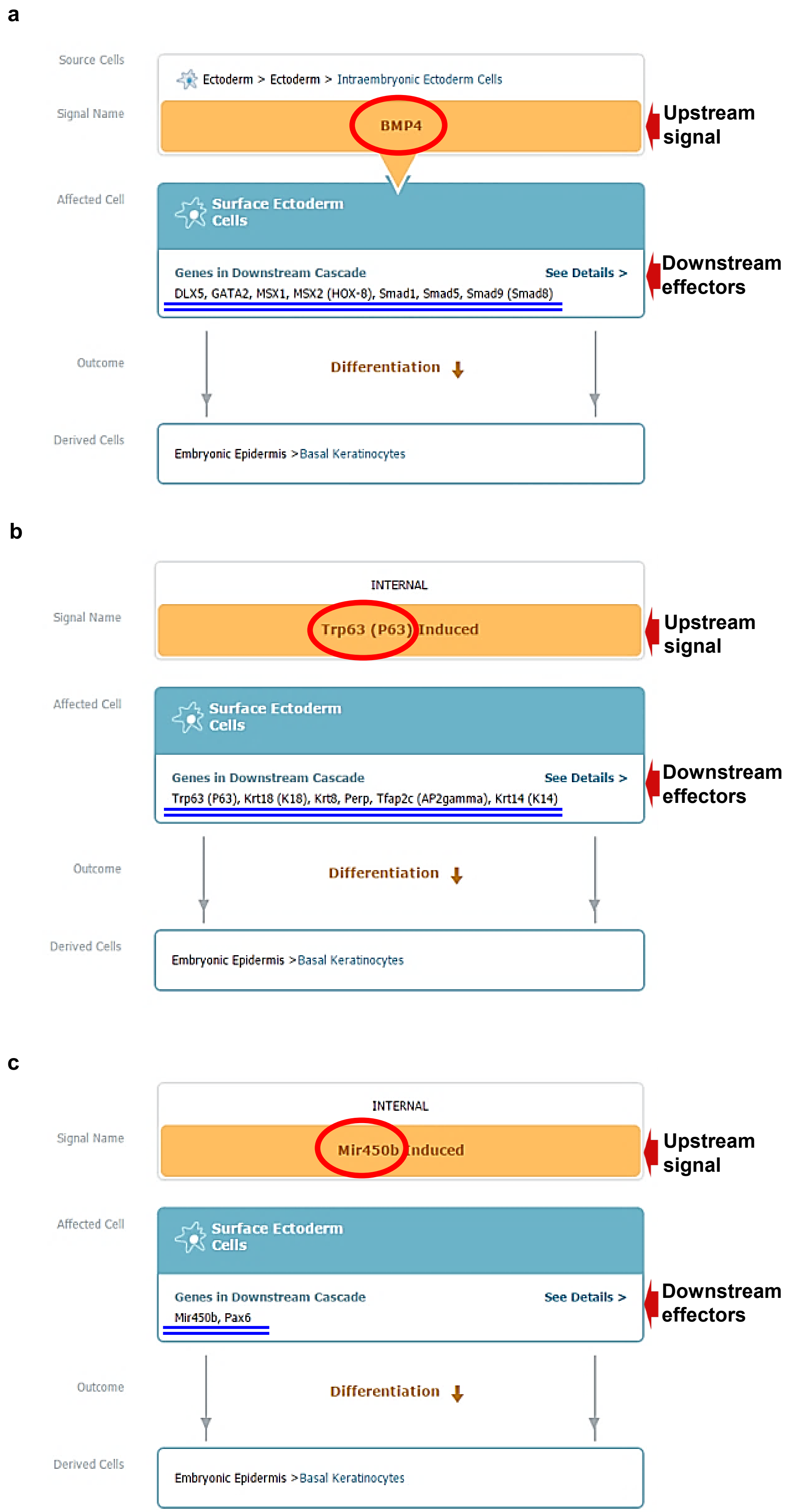
a
Source Cells
Ectoderm > Ectoderm > Intraembryonic Ectoderm Cells
Signal Name
BMP4
Upstream signal
Affected Cell
Surface Ectoderm Cells
Genes in Downstream Cascade
See Details >
DLX5, GATA2, MSX1, MSX2 (HOX-8), Smad1, Smad5, Smad9 (Smad8)
Downstream effectors
Outcome
Differentiation
Derived Cells
Embryonic Epidermis >Basal Keratinocytes
b
INTERNAL
Signal Name
Trp63 (P63) Induced
Upstream signal
Affected Cell
Surface Ectoderm Cells
Genes in Downstream Cascade
See Details >
Trp63 (P63), Krt18 (K18), Krt8, Perp, Tfap2c (AP2gamma), Krt14 (K14)
Downstream effectors
Outcome
Differentiation
Derived Cells
Embryonic Epidermis >Basal Keratinocytes
c
INTERNAL
Signal Name
Mir450b Induced
Upstream signal
Affected Cell
Surface Ectoderm Cells
Genes in Downstream Cascade
See Details >
Mir450b, Pax6
Downstream effectors
Outcome
Differentiation
Derived Cells
Embryonic Epidermis >Basal Keratinocytes

Fig.S2

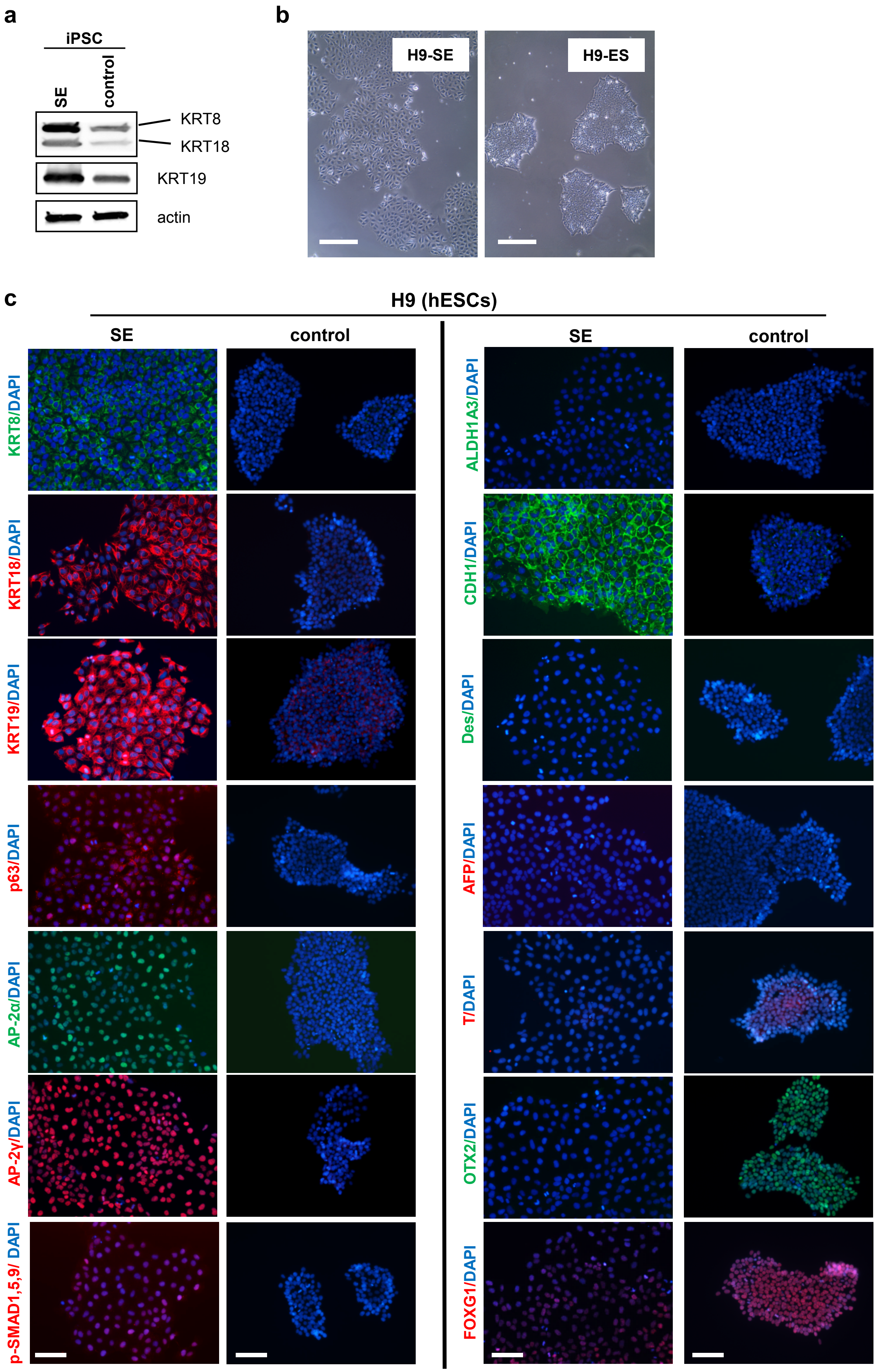
a
iPSC
SE
control
KRT8
KRT18
KRT19
actin
b
H9-SE
H9-ES
c
H9 (hESCs)
SE
control
SE
control
KRT8/DAPI
KRT18/DAPI
KRT19/DAPI
p63/DAPI
AP-2α/DAPI
AP-2γ/DAPI
p-SMAD1,5,9/ DAPI
ALDH1A3/DAPI
CDH1/DAPI
Des/DAPI
AFP/DAPI
T/DAPI
OTX2/DAPI
FOXG1/DAPI

Fig.S3

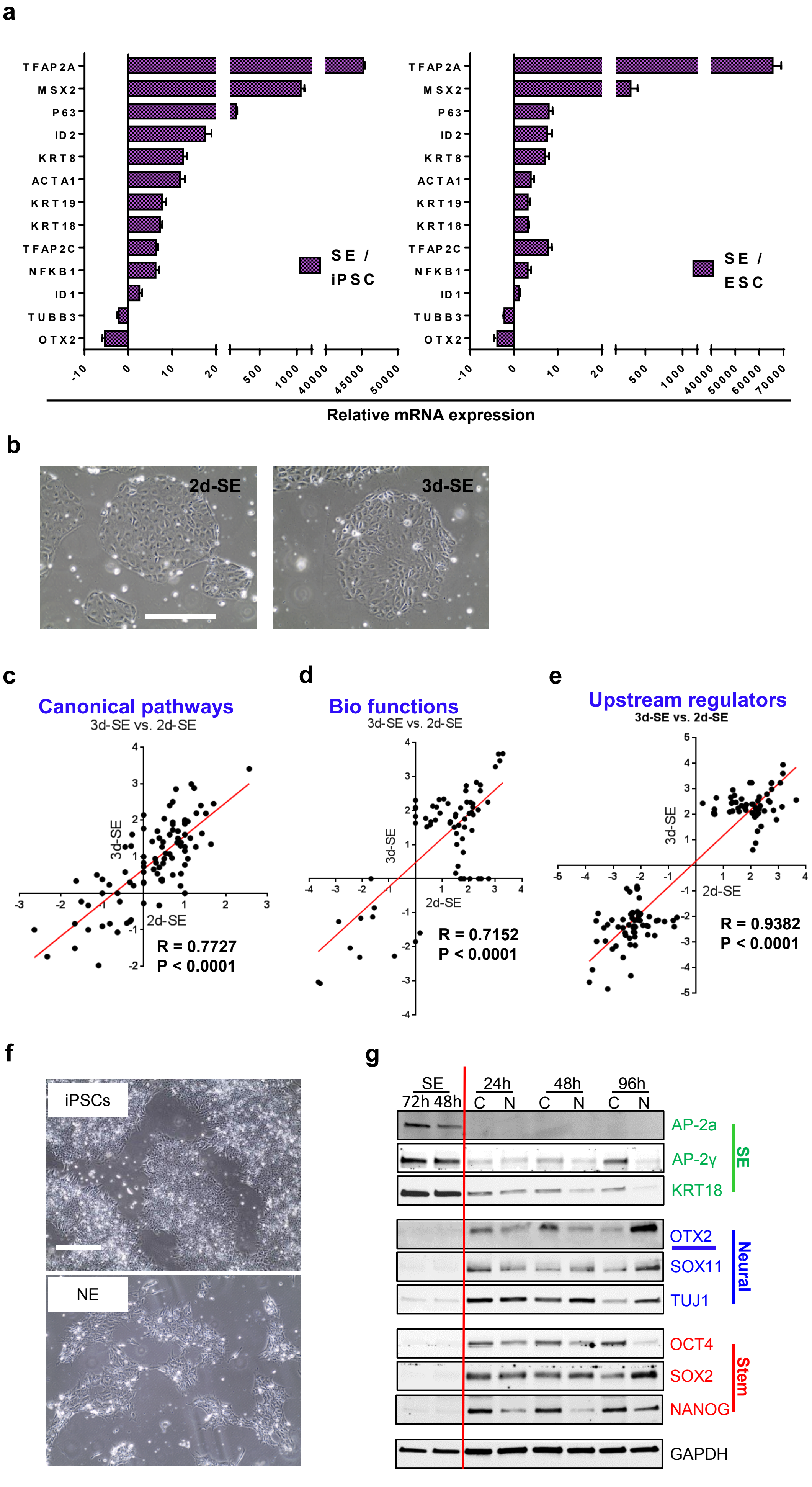
a
TFAP2A
MSX2
P63
ID2
KRT8
ACTA1
KRT19
KRT18
TFAP2C
NFKB1
ID1
TUBB3
OTX2
SE / iPSC
SE / ESC
Relative mRNA expression
b
2d-SE
3d-SE
c
Canonical pathways
3d-SE vs. 2d-SE
3d-SE
2d-SE
R = 0.7727
P < 0.0001
d
Bio functions
3d-SE vs. 2d-SE
3d-SE
2d-SE
R = 0.7152
P < 0.0001
e
Upstream regulators
3d-SE vs. 2d-SE
3d-SE
2d-SE
R = 0.9382
P < 0.0001
f
iPSCs
NE
g
SE
72h 48h
24h
48h
96h
C N C N C N
AP-2a
AP-2γ
KRT18
SE
OTX2
SOX11
TUJ1
Neural
OCT4
SOX2
NANOG
Stem
GAPDH

Fig.S4

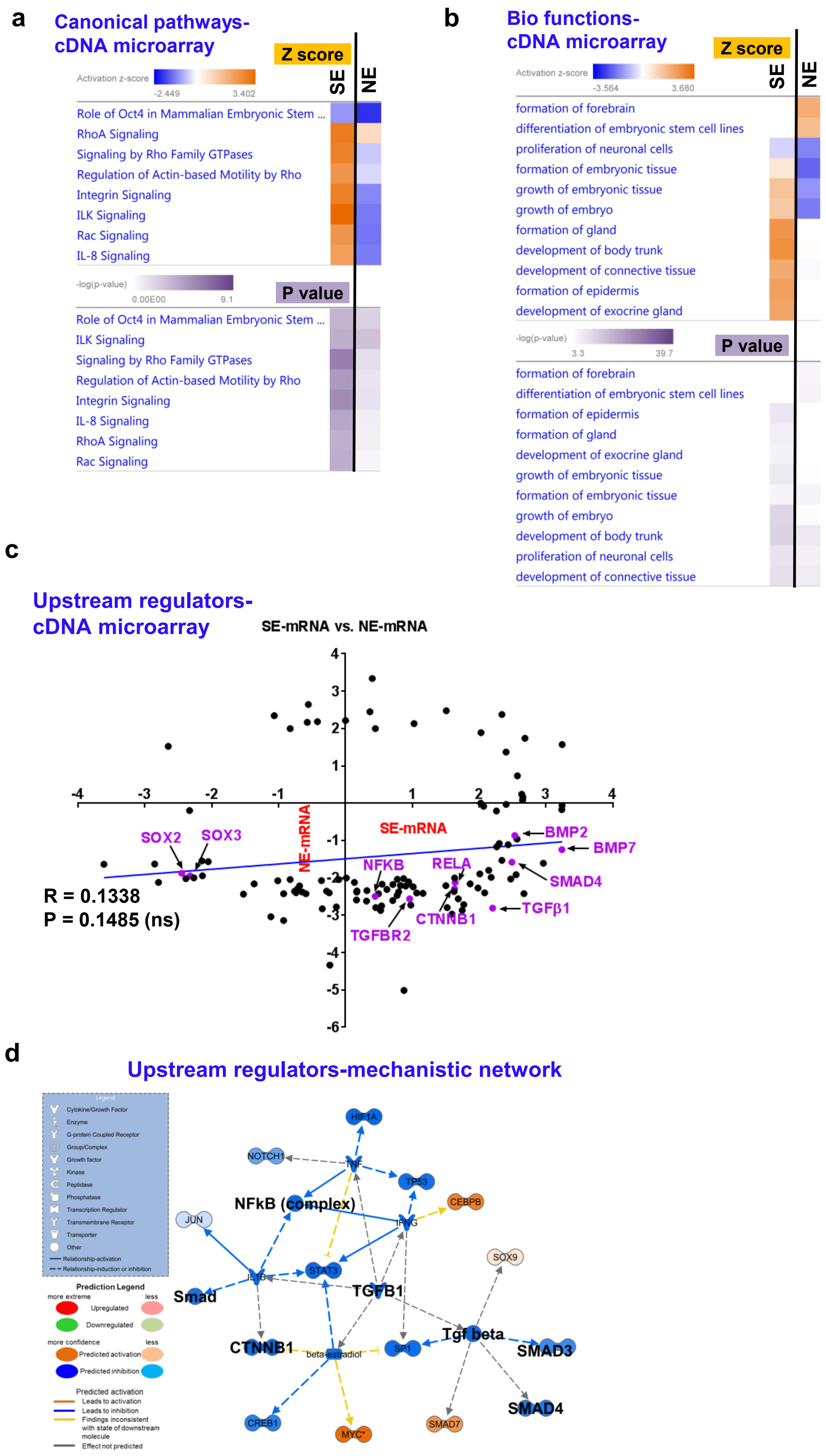
a
Canonical pathways-cDNA microarray
Z score
SE
NE
Activation z-score
-2.449
3.402
Role of Oct4 in Mammalian Embryonic Stem ...
RhoA Signaling
Signaling by Rho Family GTPases
Regulation of Actin-based Motility by Rho
Integrin Signaling
ILK Signaling
Rac Signaling
IL-8 Signaling
-log(p-value)
0.00E00
9.1
P value
Role of Oct4 in Mammalian Embryonic Stem ...
ILK Signaling
Signaling by Rho Family GTPases
Regulation of Actin-based Motility by Rho
Integrin Signaling
IL-8 Signaling
RhoA Signaling
Rac Signaling
b
Bio functions-cDNA microarray
Z score
SE
NE
Activation z-score
-3.564
3.680
formation of forebrain
differentiation of embryonic stem cell lines
proliferation of neuronal cells
formation of embryonic tissue
growth of embryonic tissue
growth of embryo
formation of gland
development of body trunk
development of connective tissue
formation of epidermis
development of exocrine gland
-log(p-value)
3.3
39.7
P value
formation of forebrain
differentiation of embryonic stem cell lines
formation of epidermis
formation of gland
development of exocrine gland
growth of embryonic tissue
formation of embryonic tissue
growth of embryo
development of body trunk
proliferation of neuronal cells
development of connective tissue
c
Upstream regulators-cDNA microarray
SE-mRNA vs. NE-mRNA
SE-mRNA
NE-mRNA
SOX2
SOX3
NFKB
RELA
BMP2
BMP7
SMAD4
TGFβ1
CTNNB1
TGFBR2
R = 0.1338
P = 0.1485 (ns)
d
Upstream regulators-mechanistic network
Legend
Cytokine/Growth Factor
Enzyme
G-protein Coupled Receptor
Group/Complex
Growth factor
Kinase
Peptidase
Phosphatase
Transcription Regulator
Transmembrane Receptor
Transporter
Other
Relationship-activation
Relationship-induction or inhibition
Prediction Legend
more extreme
less
Upregulated
Downregulated
more confidence
less
Predicted activation
Predicted inhibition
Predicted activation
Leads to activation
Leads to inhibition
Findings inconsistent with state of downstream molecule
Effect not predicted
HIF1A
NOTCH1
TNF
TP53
NFkB (complex)
CEBPB
JUN
IFNG
SOX9
IL1B
STAT3
Smad
TGFB1
CTNNB1
beta-estradiol
SP1
Tgf beta
SMAD3
SMAD4
CREB1
MYC*
SMAD7

Fig.S5

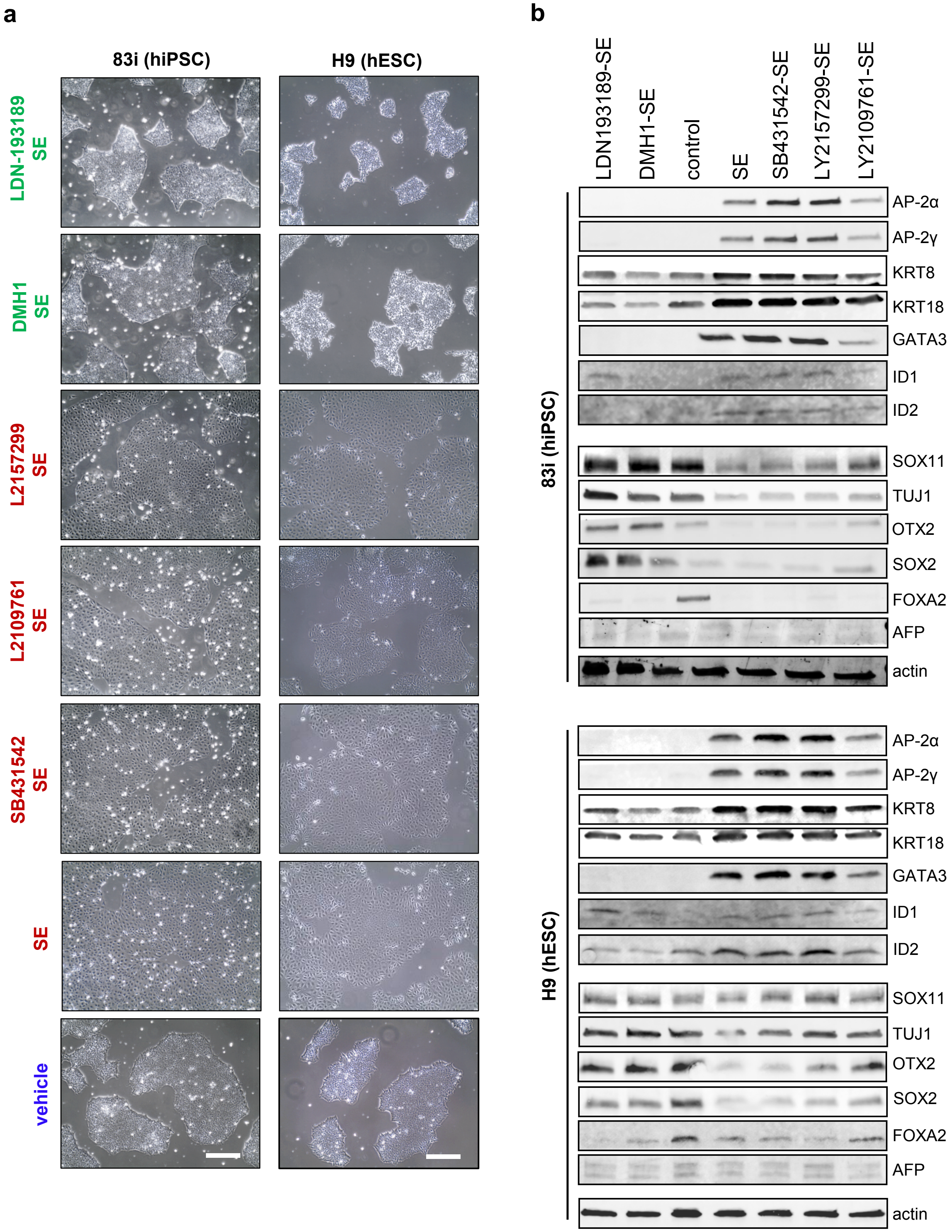
a
83i (hiPSC)
H9 (hESC)
LDN-193189 SE
DMH1 SE
L2157299 SE
L2109761 SE
SB431542 SE
SE
vehicle
b
LDN193189-SE
DMH1-SE
control
SE
SB431542-SE
LY2157299-SE
LY2109761-SE
83i (hiPSC)
H9 (hESC)
AP-2α
AP-2γ
KRT8
KRT18
GATA3
ID1
ID2
SOX11
TUJ1
OTX2
SOX2
FOXA2
AFP
actin

Fig.S6

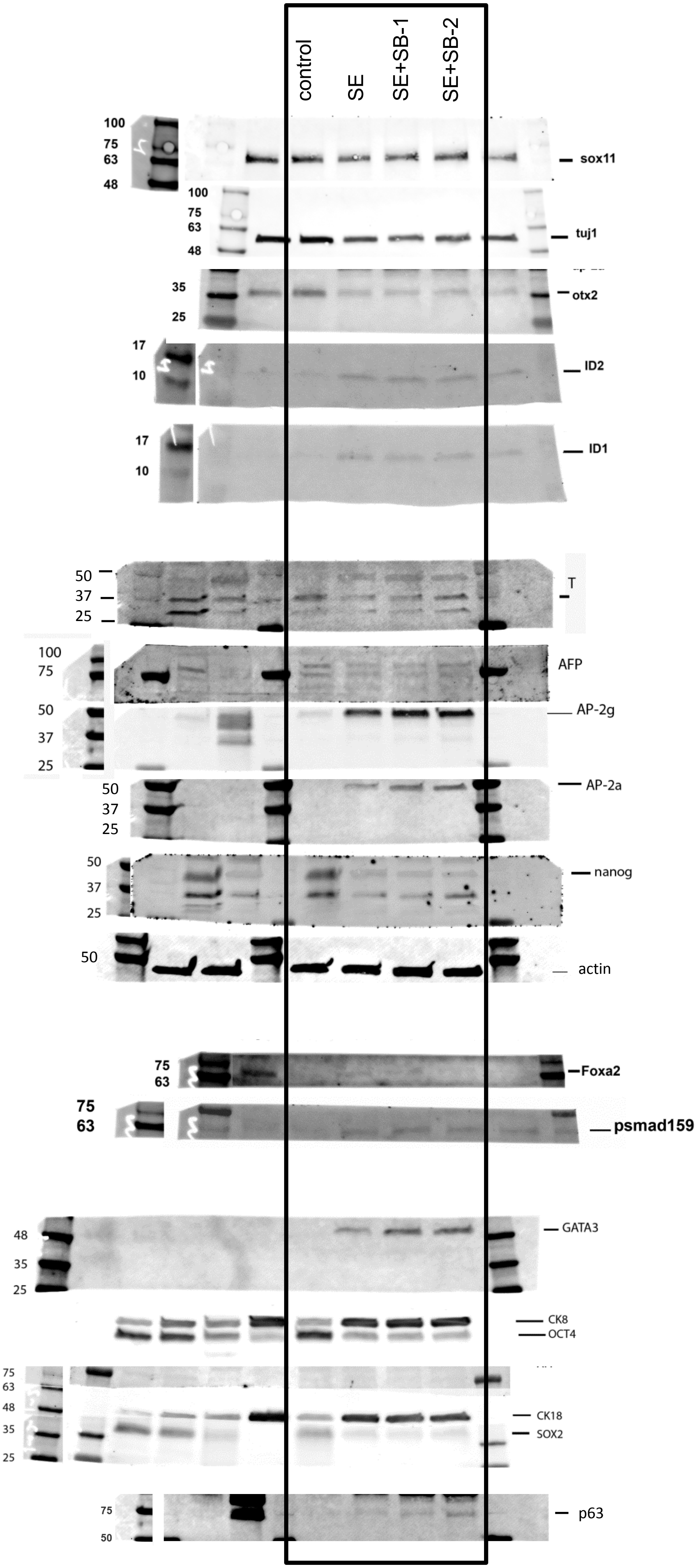

control
SE
SE+SB-1
SE+SB-2
sox11
tuj1
otx2
ID2
ID1
T
AFP
AP-2g
AP-2a
nanog
actin
Foxa2
psmad159
GATA3
CK8
OCT4
CK18
SOX2
p63
100
75
63
48
35
25
17
10
50
37
